# Risk of deep vein thrombosis (DVT) in lower extremity after total knee arthroplasty (TKA) in patients over 60 years old

**DOI:** 10.1186/s13018-023-04339-7

**Published:** 2023-11-14

**Authors:** JianQing Gao, ZhiQiang Xue, JiYue Huang, Lei Chen, JianDong Yuan, Jing Li

**Affiliations:** 1 Department of Orthopaedics, First Affiliated Hospital of Wenzhou Medical University, Wenzhou City, 325035 Zhejiang Province China; 2 Department of Orthopedics, The 900th Hospital of the People’s Liberation Army Joint Service Support Force, Fuzhou, Fujian China

**Keywords:** Total knee arthroplasty, Deep vein thrombosis, Hematocrit, Anesthesia method, Risk factors

## Abstract

**Purpose:**

There is a significant risk of DVT after TKA. We aim to evaluate the potential risk factors for postoperative DVT in the lower extremities in TKA patients over 60 years of age and provide a reference for the effective prevention of DVT.

**Methods:**

This retrospective study included patients older than 60 who underwent TKA surgery in our hospital from May 2015 to May 2022 and compared and analyzed patients' personal characteristics and clinical data with or without postoperative DVT. Logistic regression analysis was performed to determine the potential risk factors for DVT after TKA. The sensitivity and specificity of each risk factor in the diagnosis of DVT were compared by the ROC curve, and the value of this model in the diagnosis of DVT was further investigated using a multivariable combined diagnosis ROC curve model.

**Results:**

A total of 661 patients over 60 who underwent TKA were included. Preoperative Hematocrit (HCT), platelet count, anesthesia mode, postoperative D-dimer, ESR, diabetes mellitus, and other aspects of the DVT group and non-DVT group were statistically significant after TKA (*P* < 0.05). Multivariate logistics regression analysis showed that preoperative HCT, anesthesia mode, and diabetes were independent risk factors for DVT in patients over 60 years old after TKA. Compared with the univariate ROC model, the multivariable combined ROC curve analysis model has a higher diagnostic value for the diagnosis of DVT.

**Conclusion:**

DVT is common in patients over 60 years of age after TKA, and there is a multivariable influence on its pathogenesis. For patients over 60 with diabetes, neuraxial anesthesia is recommended for patients with high preoperative HCT levels, which may reduce the incidence of postoperative DVT.

## Introduction

TKA is one of the most common orthopedic surgeries to effectively treat knee joint diseases, including osteoarthritis, rheumatoid arthritis, and gouty arthritis. In recent years, the global demand for TKA surgery has been increasing, especially for middle-aged and elderly patients over 60 who receive TKA surgery to obtain better surgical results and improve their quality of life [1]. While most patients experience significant pain relief and improved functionality after TKA, some patients report poor postoperative outcomes, with no improvement or worsening symptoms. Complications contribute to patient dissatisfaction following joint arthroplasty surgery, including periprosthetic infection, DVT, anemia, and pain [2]. Among these complications, DVT is considered one of the most serious and potentially life-threatening complications following TKA, as it can lead to fatal pulmonary embolism (PE) [3].

For patients undergoing TKA, early initiation of rehabilitation exercises is crucial for the recovery of knee joint function and effectively prevents the formation of DVT [4]. Various treatment modalities have been proposed to prevent and control lower limb DVT, including risk stratification using the Caprini score and prophylactic administration of low molecular weight heparin [5]. These methods have demonstrated varying degrees of efficacy, but there are still cases where DVT occurs despite appropriate treatment.

Anesthesia is crucial in the TKA surgical process and postoperative pain management. There is no gold standard for the most suitable anesthesia method for TKA, as it depends on the skills and preferences of the anesthesiologist. In recent years, it has been reported that different anesthesia methods used in TKA may affect the occurrence of postoperative complications, such as respiratory depression, pulmonary infection, PE, and heart disease [6], which provides a new direction for orthopedic research on the prevention of postoperative DVT. In addition, most middle-aged and elderly patients who need TKA treatment are often accompanied by chronic diseases such as hypertension and diabetes, and there has been no clear research on the optimal anesthesia method for these patients. Therefore, the purpose of this study is to identify the risk factors for DVT after TKA in elderly patients over 60 years old, explore the influence of different anesthesia methods on postoperative DVT of lower limbs in middle-aged and elderly TKA patients, and provide reference and clinical guidance for the prevention of DVT of lower limbs.

## Methods

This retrospective study was approved by the Ethics Review Committee of the First Affiliated Hospital of Wenzhou Medical University, and informed consent was obtained from all participants. The study included patients aged 60 and above with knee osteoarthritis who underwent unilateral TKA at the First Affiliated Hospital of Wenzhou Medical University between May 2015 and May 2022. All procedures in this study were conducted according to the principles outlined in the Helsinki Declaration.

All data were obtained from the electronic medical record system of the medical center. We included patients who underwent unilateral TKA at our hospital between May 2015 and May 2022. The inclusion criteria for this study were as follows: (1) meeting the surgical criteria for TKA as the first procedure; (2) a Tourniquet was used to control bleeding during the operation. (3) Age 60 years and above; (4) The perioperative data entirely case data (pre- and postoperative testing, at least two times of vascular Doppler ultrasound (DU) examination were performed after operation, and anesthetic information); (5) Preoperative lower limb vascular DU confirming the absence of thrombosis in both lower limbs; (6) Inclusion of patients with calf muscle vein thrombosis. The exclusion criteria were as follows: (1) Patients with previously diagnosed lower extremity DVT or lower extremity varicose veins; (2) Presence of any condition requiring anticoagulant or antiplatelet therapy (For example, post-cardiac valve surgery, atrial fibrillation, and coronary stent placement); (3) The cases of failure of NA and temporary change to general anesthesia (GA) were excluded; (4) Exclusion of patients with hemophilic arthritis due to abnormal coagulation function, as well as patients with rheumatoid arthritis, as the abnormal inflammatory factors can affect the occurrence and development of DVT; (5) exclusion of patients with malignancy, pathological fractures, allergy to anticoagulant drugs, thrombocytopenia, recent gastrointestinal bleeding, or a history of stroke within 3 months, as these conditions can potentially influence the development of DVT; (6) exclusion of patients with incomplete medical records.

The diagnostic criteria for lower limb DVT were based on relevant clinical practice guidelines [7]. Thromboembolic risk assessment was routinely performed for all admitted patients. Patients without contraindications for anticoagulation received basic thromboprophylaxis, including chemoprophylaxis with low molecular weight heparin (LMWH) at a dose of 3800 IU/0.4 ml once daily, along with mechanical prophylaxis. Before and following surgery, patients were actively encouraged to engage in knee joint functional rehabilitation exercises and early mobilization. Pre- and postoperative color DU examinations of the affected lower limb veins were performed by experienced radiologists to diagnose the occurrence of DVT. The diagnosis of DVT was based on assessing vessel and blood flow velocity using ultrasound [8], with lower limb deep veins being checked every 2–3 days. Venography was performed when necessary to confirm DVT. Based on the DU results, patients were classified into DVT and non-DVT groups. If DU confirmed DVT, physical prophylaxis was discontinued, and inferior vena cava filters were considered if necessary to prevent fatal PE.

The collected data included detailed demographic information, comorbidities, personal medical history, smoking and alcohol history, pre-and postoperative laboratory tests (including complete blood count, liver and kidney function, coagulation function, and D-dimer), preoperative preparation time, and surgical factors (operative time, anesthesia method).

### Statistical analysis

Statistical analysis was performed using SPSS 26.0 software (IBM, New York, USA). Quantitative data are presented as mean ± standard deviation, while qualitative data are presented as numbers and percentages. Depending on the data type and distribution, the independent samples *t*-test or Mann–Whitney test was used for comparing quantitative variables, and the chi-square test or Fisher's exact test was used for comparing qualitative variables. Variables with a *P* value < 0.05 in the univariate logistic regression analysis were entered into the multivariate logistic regression analysis to identify the risk factors for DVT occurrence in TKA patients aged 60 and above. Receiver operating characteristic (ROC) curves were constructed using MedCalc 20.0 software. The optimal cutoff values for each variable were determined based on the Youden index, and the diagnostic value of the multivariate logistic regression model in predicting DVT occurrence in TKA patients aged 60 and above was evaluated using the ROC curve. This study's *P* value < 0.05 was considered statistically significant for differences.

## Results

**Fig. 1:**
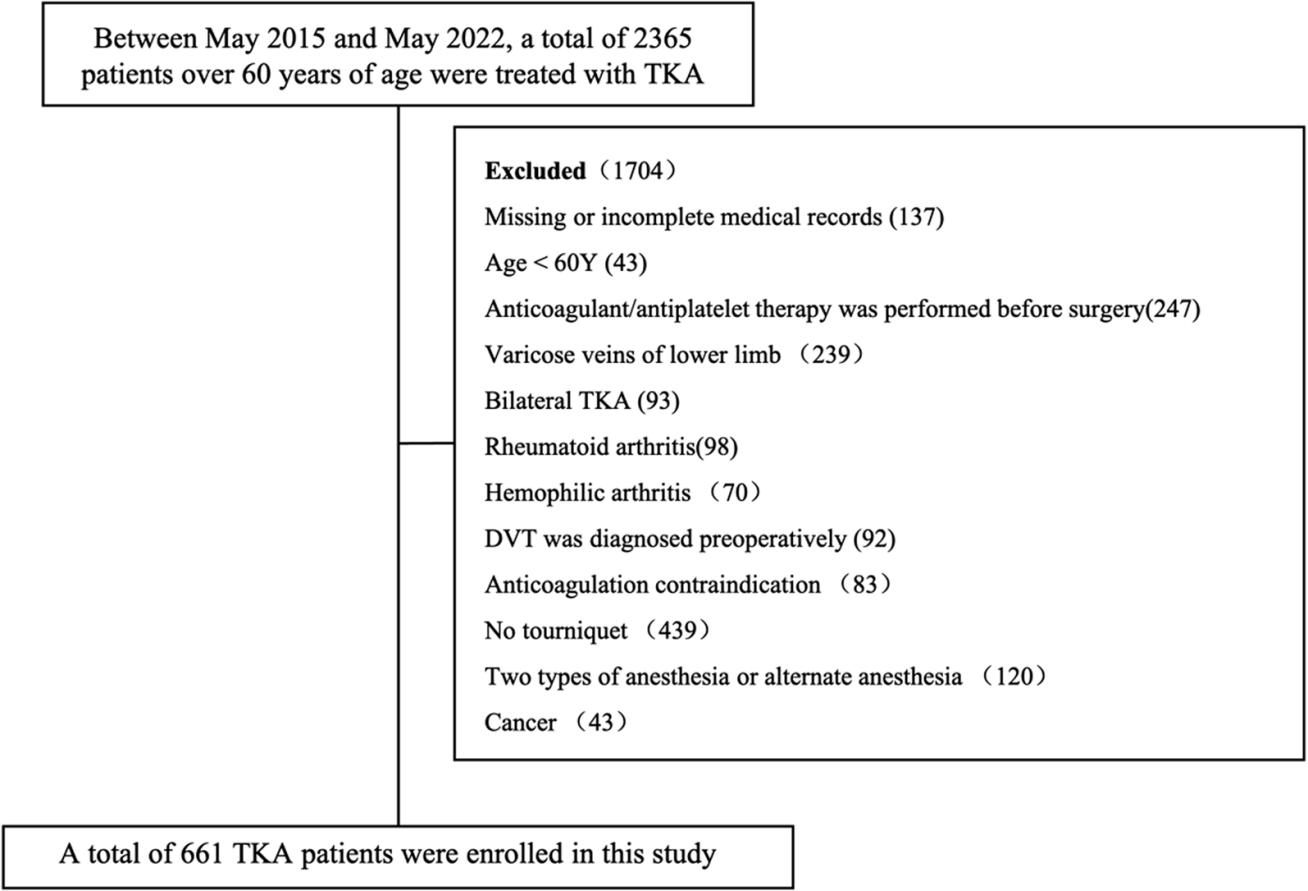
Exclusion criteria and the number of studies were included in this study

A total of 661 patients aged 60 and above who underwent TKA were included in this study. Detailed information on excluded cases is shown in Fig. 1. Among them, 207 patients developed DVT, resulting in a DVT occurrence rate of 31.3% in TKA patients. The average age of patients who developed DVT was 70.13 ± 7.05 years, with 65 males and 142 females. In the univariate analysis, significant differences (*P* < 0.05) were observed between patients with and without DVT in variables such as preoperative HCT, platelet count (PLT), postoperative D-dimer and erythrocyte sedimentation rate (ESR) levels, presence of diabetes, and anesthesia method. However, there were no statistically significant differences in age, smoking, alcohol consumption, body mass index (BMI), preoperative preparation time, blood lipids, albumin (ALB) levels, or duration of surgery times. Detailed characteristics of the two groups are presented in Table 1.

**Table 1:** Demographic characteristics and univariate analysis of DVT after TKA surgery

Variables	DVT *n* = 207	No-DVT *n* = 454	*P* value
Number of patients	207 (31.3%)	454 (68.7%)	–
Gender (*n*, %)			0.657
Male	65 (31.4%)	153 (33.7%)	
Female	142 (68.5%)	301 (66.3%)	
Age (mean ± SD)	70.13 ± 7.05	69.33 ± 7.15	0.178
BMI (mean ± SD)	25.38 ± 3.77	25.75 ± 13.27	0.694
Side (*n*, %)			0.197
Left side	81 (39.1%)	207 (45.6%)	
Right side	126 (60.8%)	247 (54.4%)	
Smoking (*n*, %)	15 (7.2%)	51 (11.2%)	0.113
Alcohol (*n*, %)	17 (8.2%)	54 (11.9%)	0.156
Preoperative period			
Preoperative time (days)	3.01 ± 1.42	3 ± 1.48	0.937
Triglyceride (mmol/L)	2.29 ± 1.47	2.23 ± 1.34	0.579
Cholesterol (mmol/L)	5.34 ± 2.01	5.30 ± 1.06	0.746
Fibrinogen (g/L)	3.43 ± 0.77	3.41 ± 0.76	0.763
Hb (g/L)	128.07 ± 13.36	129.94 ± 13.86	0.104
HCT (L/L)	0.45 ± 0.05	0.39 ± 0.04	0.018
ALB (g/L)	39.52 ± 3.31	39.51 ± 3.16	0.97
PLT (10^9/L)	247.02 ± 64.78	233.29 ± 65.32	0.012
D-dimer (mg/L)	0.70 ± 1.04	1.10 ± 10.09	0.574
Intraoperative period			
Duration of surgery times	82.03 ± 20	84.38 ± 22.59	0.248
Anesthesia (*n*, %)			< 0.001
GA	114 (55.1%)	200 (44.1%)	
NA	93 (44.9%)	254 (55.9%)	
Postoperative period			
Triglyceride (mmol/L)	1.24 ± 0.52	1.40 ± 0.94	0.058
Cholesterol (mmol/L)	4.71 ± 1.03	4.63 ± 1.05	0.514
Fibrinogen (g/L)	4.32 ± 1.32	4.16 ± 1.26	0.201
Hb (g/L)	119.38 ± 14.01	123.48 ± 48.67	0.236
ALB (g/L)	35.02 ± 2.77	35.28 ± 3.54	0.364
PLT (10^9^/L)	213.89 ± 61.39	209.60 ± 58.33	0.39
D-dimer (mg/L)	2.95 ± 2.48	3.88 ± 4.30	0.048
ESR (mm/h)	8.20 ± 3.90	29.90 ± 19.12	0.018
HCT	0.37 ± 0.04	0.35 ± 0.04	0.175
Preoperative comorbidities			
Diabetes (*n*, %)	36 (17.4%)	55 (12.1%)	< 0.001
Hypertension (*n*, %)	90 (43.5%)	176 (38.8%)	0.252

*BMI* body mass index, *Hb* hemoglobin, *HCT* hematocrit, *ALB* albumin, *PLT* platelets, *ESR* erythrocyte sedimentation rate, *GA* general anesthesia, *NA* neuraxial anesthesia

The factors with statistically significant differences in the univariate analysis were included in a univariate logistic regression analysis. The study revealed that preoperative HCT, diabetes, and anesthesia methods were potential risk factors for DVT in TKA patients. By controlling for confounding factors and including the variables above in a multivariate logistic regression analysis model, it was found that preoperative HCT (OR 1.72, 95% CI 1.54–2.89, *P* = 0.04), diabetes (OR 2.33, 95% CI 1.47–3.67, *P* < 0.001), and anesthesia method (OR 1.79, 95% CI 1.28–2.51, *P* = 0.001) were independent risk factors for DVT occurrence in TKA patients aged 60 and above (Table 2).

**Table 2:** Risk factors of DVT after Univariate and Multivariable logistics regression analyzed TKA surgery

Variable	Univariate		Multivariable	
	OR (95%CI)	*P*	OR (95%CI)	*P*
Preoperative HCT	1.69 (1.42–2.61)	0.018	1.72 (1.54–2.89)	0.04
Diabetes	2.28 (1.46–3.57)	< 0.001	2.33 (1.47–3.67)	< 0.001
Anesthesia	1.76 (1.26–2.44)	< 0.001	1.79 (1.28–2.51)	0.001

*HCT* Hematocri

**Fig. 2:**
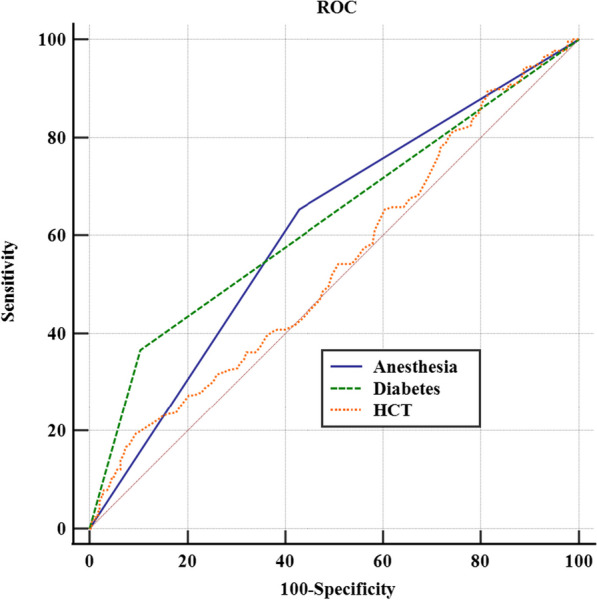
The AUCs of different variables represent the ability of each risk factor to differentiate the risk of DVT

**Fig. 3:**
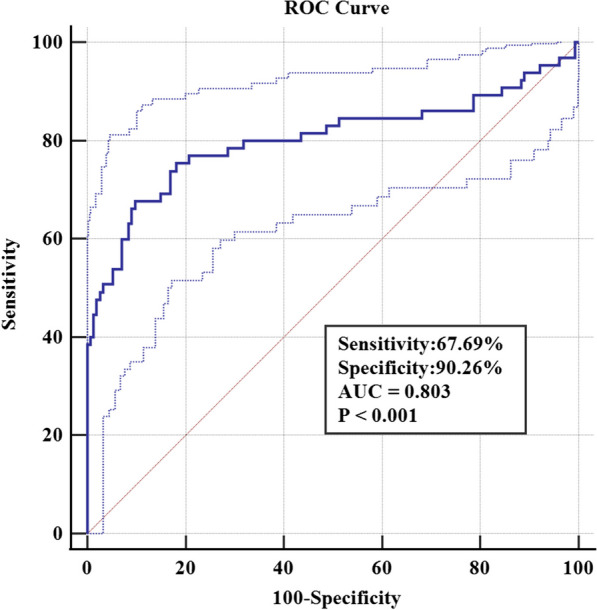
ROC curve analysis of multivariable combined diagnosis

ROC curve analysis was performed to evaluate the area under the curve (AUC), sensitivity, and specificity of preoperative HCT, diabetes, and anesthesia (Table 3). The AUC for preoperative HCT was 0.587 (95% CI 0.550–0.622, *P* < 0.001), with a sensitivity of 67.59% and specificity of 71.40%. The optimal cutoff point was determined to be > 0.4. For diabetes, the AUC was 0.631 (95% CI 0.593–0.668, *P* < 0.001), with a sensitivity of 36.57% and specificity of 89.66%. The AUC for the anesthesia method was 0.612 (95% CI 0.573–0.649, *P* < 0.001), with a specificity of 60.28% and sensitivity of 57.08% (Fig. 2). In the multivariate analysis, the combined diagnosis using the factors above yielded an AUC of 0.803 (95% CI 0.744–0.854, *P* < 0.001), with a sensitivity of 67.69% and specificity of 90.26% (Fig. 3).

**Table 3:** AUC values for each risk factor under the ROC curve

Variable	AUC	95%CI		*P*
		Lower limit	Upper limit	
Preoperative HCT	0.587	0.550	0.622	< 0.001
Anesthesia	0.612	0.573	0.649	< 0.001
Diabetes	0.631	0.593	0.668	< 0.001

*HCT* Hematocrit

## Discussion

The surgical volume of TKA has significantly increased due to population aging, obesity, and advancements in joint prosthesis quality [9]. On the one hand, as population activity and exercise-related injuries continue to rise, osteoarthritis has been notable among middle-aged and elderly individuals below 65. Moreover, younger age and improved preoperative health conditions have been linked to more favorable outcomes following TKA, leading to further escalation in knee arthroplasty rates within this demographic [10]. In this study, the incidence of DVT after TKA was 31.3%, higher than the previously reported rate of 16.8% [11]. The patients included in the present study are middle-aged and elderly patients who underwent more rigorous and frequent DU examinations of lower extremity vessels after surgery. In addition, we included patients with isolated muscle-vein thrombosis of the calf because muscle-vein thrombosis can progress to a significant DVT of the lower extremity, and shedding of a giant muscle-vein thrombus can lead to pulmonary embolism [12].

Current screening for DVT mainly relies on strict DU diagnosis, but limited by resources, it is impossible to implement dynamic monitoring of thrombus in each patient. A retrospective study of 1848 trauma deaths found that ISS (Iniurv Severity Score) < 16 patients with lower-extremity injuries were at risk for fatal PE after discharge, but only 8% had venous thromboembolism diagnosed by DU before death [13]. We believe there are still more undiagnosed DVT cases, and the incidence of DVT will be further increased if the rate of DU examination is increased. Therefore, for middle-aged and elderly patients, we should take more active measures based on the original DVT prevention, such as the combination of multiple physical prevention (ankle pump exercise combined with intermittent air pressure pump or elastic socks), regular monitoring of coagulation function and vascular DU, active prevention of postoperative pain, and early postoperative knee joint functional exercise.

HCT level is one of the critical determinants of blood viscosity. It is associated with increased blood viscosity, decreased venous return, and increased exposure of endothelial cells to platelets and coagulation factors [14]. Therefore, theoretically, patients with HCT levels above the normal range are more likely to develop DVT.

The study by Vaya found a significantly higher rate of DVT in patients with HCT, more significant than 45% [15]. In another study, Yu-Min Zhang [16] found a nonlinear association between HCT levels at admission and preoperative DVT in patients with hip fractures. That HCT at admission was a risk factor for preoperative DVT, and when HCT was 33.5%, the severity of low hematocrit was not associated with preoperative DVT. There are few studies on the relationship between HCT and DVT after TKA. This study found that high preoperative HCT level is an independent risk factor for DVT in elderly patients over 60 years old after TKA, increasing the risk of postoperative DVT by 1.72 times. To more accurately evaluate the relationship between HCT levels and postoperative DVT, we used the ROC curve to determine the optimal cutoff point as 0.40. Patients with a preoperative HCT > 0.4 are considered at high risk for postoperative DVT after TKA. Related studies have explained that increased HCT may benefit clot formation by increasing circulating platelets and promoting the residence time of clotting factors near the vascular endothelium [17]. Slow blood loss during the operation leads to the loss of various cellular components, including red blood cells. Without strict fluid management during the operation, it is easy to cause body fluid to infiltrate the tissue space, resulting in blood concentration, which manifests as increased HCT and ultimately increases the risk of DVT [18, 19]. Therefore, for patients with high preoperative HCT levels, preoperative fluid volume should be increased to dilute each cell component, reduce intraoperative bleeding, strict fluid management, and monitor whether bleeding continues in the joint cavity after surgery to prevent the increase of DVT risk due to hemoconcentration.

In order to control intraoperative bleeding and shorten the operation time, some doctors choose to use tourniquets. However, they also face safety issues, such as increasing the incidence of postoperative DVT and exacerbating postoperative pain and swelling in the short term [20]. The use of a tourniquet is not strictly prescribed, and current studies suggest that early tourniquet release in patients undergoing TKA reduces the incidence of DVT without increasing the incidence of complications [21].

In this study, the risk of DVT after TKA in patients with diabetes mellitus was significantly increased. Diabetes mellitus was an independent risk factor for DVT after TKA, and the risk of diabetic patients was 2.33 times higher than non-diabetic patients. The results of our study are similar to those of Wang [22], suggesting that patients with diabetes have a 2.76 times higher risk of developing DVT within 14 days after TKA than those without diabetes. There are often a variety of abnormal metabolic states in diabetic patients. These metabolic abnormalities can affect endothelial cells through oxidative stress, inflammation, protein glycosylation, and other ways, leading to vascular intimal damage, endothelial dysfunction, increased platelet activity, and coagulation system imbalance [23]. Therefore, the development of DVT may be exacerbated by the combined effects of surgery and diabetes.

A growing body of evidence suggests that the choice of perioperative anesthesia in TKA may affect the risk of postoperative complications and mortality [24]. Memtsoudis [25] compared GA and NA and determined whether the choice of anesthesia impacted perioperative outcomes. In the TKA subgroup, GA was associated with higher rates of PE, pneumonia, systemic infection, acute renal failure, and 30-day overall mortality. This study found that GA after TKA was associated with a higher risk of DVT (OR 1.79, 95%CI 1.28–2.51, *P* = 0.001). This result is similar to the study's conclusion by Callaghan [26]. The choice of anesthesia method is an independent risk factor for short-term complications after TKA. The current study suggests that the type of anesthesia does not only affect the incidence of DVT after TKA. Moucha [27] compared the incidence of postoperative complications in 6133 patients who underwent hip fracture surgery under spinal or general anesthesia. The number of patients with postoperative DVT increased significantly. The effect of GA drugs on the central nervous system can inhibit sympathetic nerve activity, leading to vasodilation and a decrease in cardiac output [28], slowing down blood flow in the lower limb veins and increasing the possibility of blood retention.

On the other hand, narcotic drugs can interfere with the synthesis and metabolism of clotting factors and cause platelet activation and aggregation, causing the blood to agglutinate more quickly, making it easier to attach to the inner wall of blood vessels and form thrombosis [29, 30]. Current studies suggest that the mechanism of DVT caused by GA affecting the blood circulation system is complex and diverse. More research is needed to investigate the potential mechanisms of these findings. Therefore, reducing the side effects of anesthetic drugs to effectively ensure intraoperative analgesia through NA may reduce the incidence of postoperative DVT [31].

We used ROC curves to detect independent risk factors to predict the value of postoperative DVT. According to the ROC curve, we found that the AUC of each risk factor did not reach the ideal level, suggesting that the occurrence of DVT is multivariable and complex and that the accuracy of predicting the occurrence of DVT after TKA by a single factor is insufficient. Each risk factor was put into a multivariate logistic regression model. After ROC curve analysis, the AUC of the model was significantly increased to 0.803, the specificity was 90.26%, and the sensitivity was 67.69%. Multivariate ROC curve analysis showed that the model had a high predictive value for the occurrence of DVT after TKA in patients over 60 years old. It should be pointed out that the results of this study will help us to identify the potential population of middle-aged and elderly patients who may develop DVT after TKA, and more stringent perioperative DVT prevention and treatment combined with anesthesia during the operation is expected to reduce the incidence of postoperative DVT further.

There are some limitations to this study. First, this is a retrospective study, limited by a single center and small case sample size, and the results of this study should be treated with caution. Second, for patients with diabetes, we did not distinguish the types, such as type 1 or type 2, and we did not routinely detect HBA1c. Because our study was a retrospective design, we could not collect most of the data on HBA1c, so we could not include these indicators in the analysis. Third, this study lacks follow-up and dynamic observation. Some patients after TKA may develop DVT after discharge, and the data for this part cannot be obtained, resulting in the survivor effect. In the future, larger samples and multi-center studies are needed to further explore the risk factors for DVT in patients over 60 years of age after TKA surgery to provide reliable evidence for preventing DVT.

## Conclusion

In summary, this study confirms that the incidence of DVT after TKA is higher in patients over 60 years of age and that diabetes, preoperative HCT, and type of anesthesia may be independent risk factors for postoperative DVT. Therefore, for patients over 60 years old with diabetes who plan to undergo TKA, it is recommended to actively adjust the preoperative HCT level of the patient and choose NA during the operation, which may help to reduce the level of postoperative DVT.

## Data Availability

Due to hospital management regulations and related policies, the data of this study cannot be published. If the journal needs it, it can be provided, but it cannot be published.

## References

[1] Anis HK, Strnad GJ, Klika AK (2020). Developing a personalized outcome prediction tool for knee arthroplasty. Bone Joint J.

[2] Arshi A, Leong NL, D'Oro A (2017). Outpatient total knee arthroplasty is associated with higher risk of perioperative complications. J Bone Joint Surg Am.

[3] Merli GJ (2001). Duration of deep vein thrombosis and pulmonary embolism prophylaxis after joint arthroplasty. Med Clin N Am.

[4] Fortier LM, Rockov ZA, Chen AF (2021). Activity recommendations after total hip and total knee arthroplasty. J Bone Joint Surg Am.

[5] Hayssen H, Cires-Drouet R, Englum B (2022). Systematic review of venous thromboembolism risk categories derived from Caprini score. J Vasc Surg Venous Lymphat Disord.

[6] Wilson JM, Farley KX, Erens GA (2019). General vs spinal anesthesia for revision total knee arthroplasty: Do complication rates differ?. J Arthroplasty.

[7] Tritschler T, Kraaijpoel N, Le Gal G (2018). Venous thromboembolism: advances in diagnosis and treatment. JAMA.

[8] Mantoni M (2001). Ultrasound of limb veins. Eur Radiol.

[9] Kurtz S, Ong K, Lau E (2007). Projections of primary and revision hip and knee arthroplasty in the United States from 2005 to 2030. J Bone Joint Surg Am.

[10] Losina E, Thornhill TS, Rome BN (2012). The dramatic increase in total knee replacement utilization rates in the United States cannot be fully explained by growth in population size and the obesity epidemic. J Bone Joint Surg Am.

[11] Wang X, Xi H, Geng X (2023). Artificial intelligence-based prediction of lower extremity deep vein thrombosis risk after knee/hip arthroplasty. Clin Appl Thromb Hemost.

[12] Schwarz T, Buschmann L, Beyer J (2010). Therapy of isolated calf muscle vein thrombosis: a randomized, controlled study. J Vasc Surg.

[13] Kalkwarf KJ, Yang Y, Mora S (2023). The silent killer: previously undetected pulmonary emboli that result in death after discharge. Injury.

[14] Alexy T, Detterich J, Connes P (2022). Physical properties of blood and their relationship to clinical conditions. Front Physiol.

[15] Vayá A, Falcó C, Simó M (2007). Influence of lipids and obesity on haemorheological parameters in patients with deep vein thrombosis. Thromb Haemost.

[16] Li DY, Lu DX, Yan T (2023). The association between the hematocrit at admission and preoperative deep venous thrombosis in hip fractures in older people: a retrospective analysis. J Clin Med.

[17] Hellem AJ, Borchgrevink CF, Ames SB (1961). The role of red cells in haemostasis: the relation between haematocrit, bleeding time and platelet adhesiveness. Br J Haematol.

[18] Baron DM, Metnitz PG, Fellinger T (2016). Evaluation of clinical practice in perioperative patient blood management. Br J Anaesth.

[19] Keating EM, Meding JB (2002). Perioperative blood management practices in elective orthopaedic surgery. J Am Acad Orthop Surg.

[20] Palanne R, Rantasalo M, Vakkuri A (2023). Testing of a predictive risk index for persistent postsurgical pain on patients undergoing total knee arthroplasty: a prospective cohort study. Eur J Pain.

[21] Zan P, Mol MO, Yao JJ (2017). Release of the tourniquet immediately after the implantation of the components reduces the incidence of deep vein thrombosis after primary total knee arthroplasty. Bone Joint Res.

[22] Wang S, Zhao Y (2013). Diabetes mellitus and the incidence of deep vein thrombosis after total knee arthroplasty: a retrospective study. J Arthroplasty.

[23] Kaur R, Kaur M, Singh J (2018). Endothelial dysfunction and platelet hyperactivity in type 2 diabetes mellitus: molecular insights and therapeutic strategies. Cardiovasc Diabetol.

[24] Moucha CS, Weiser MC, Levin EJ (2016). Current strategies in anesthesia and analgesia for total knee arthroplasty. J Am Acad Orthop Surg.

[25] Memtsoudis SG, Sun X, Chiu YL (2013). Perioperative comparative effectiveness of anesthetic technique in orthopedic patients. Anesthesiology.

[26] Pugely AJ, Martin CT, Gao Y (2013). Differences in short-term complications between spinal and general anesthesia for primary total knee arthroplasty. J Bone Joint Surg Am.

[27] Fields AC, Dieterich JD, Buterbaugh K (2015). Short-term complications in hip fracture surgery using spinal versus general anaesthesia. Injury.

[28] Zhong H, Wang Y, Wang Y (2019). Comparison of the effect and clinical value in general anesthesia and combined spinal-epidural anesthesia in elderly patients undergoing hip arthroplasty. Exp Ther Med.

[29] Durant TJ, Dwyer CR, McCarthy MB (2017). Protective nature of platelet-rich plasma against chondrocyte death when combined with corticosteroids or local anesthetics. Am J Sports Med.

[30] Liu D, Sun C, Zhang X (2021). Influence of epidural anesthesia and general anesthesia on thromboembolism in patients undergoing total knee arthroplasty. Am J Transl Res.

[31] Lavand'homme PM, Kehlet H, Rawal N (2022). Pain management after total knee arthroplasty: PROcedure SPEcific Postoperative Pain ManagemenT recommendations. Eur J Anaesthesiol.

